# Improving UV Stability of SiO_2_/SiN_x_-Passivated Silicon Photodiodes Through Shallow Junction Implantation and Oxide Regrowth

**DOI:** 10.3390/s26133991

**Published:** 2026-06-24

**Authors:** Michael N. Getz, Ozhan Koybasi, Fredrik Edhborg, Ørnulf Nordseth, Steven Hesse, Tobias Pohl, Marco Povoli, Stefan Källberg, Lutz Werner, Erkki Ikonen, Jarle Gran

**Affiliations:** 1 Smart Sensors and Microsystems, SINTEF Digital, 0314 Oslo, Norway; ozhan.koybasi@sintef.no (O.K.); marco.povoli@sintef.no (M.P.); 2 RISE Research Institutes of Sweden, 50115 Borås, Swedenstefan.kallberg@ri.se (S.K.); 3 Department of Solar Energy Materials and Technology, Institute for Energy Technology, 2027 Kjeller, Norway; 4 Physikalisch-Technische Bundesanstalt, 10587 Berlin, Germanytobias.pohl@ptb.de (T.P.);; 5 Metrology Research Institute, Aalto University, 02150 Espoo, Finland; erkki.ikonen@aalto.fi; 6 The Norwegian Metrology Service (Justervesenet), 2027 Kjeller, Norway; jag@justervesenet.no

**Keywords:** induced-junction photodiodes, ultraviolet photodiodes, silicon photodiodes, SiO_2_/SiN_x_ passivation, shallow ion implantation, UV stability

## Abstract

Induced-junction silicon photodiodes based on SiO_2_/SiN_x_ surface passivation are attractive for high-accuracy radiometry, but their use in the deep ultraviolet is limited by UV-induced degradation of the dielectric stack. In this work, we investigate the degradation of SiO_2_/SiN_x_-passivated p-type silicon photodiodes under UV irradiation and evaluate strategies for improving stability through shallow implanted junctions and oxide processing. Capacitance–voltage measurements on MIS capacitors and lifetime measurements on symmetrically passivated wafers show that UV exposure causes a rapid reduction in effective dielectric charge and carrier lifetime, followed by saturation at higher dose, consistent with filling of a finite population of electrically active trap states. Induced-junction photodiodes exhibit rapid photocurrent loss at 222 nm and, in some cases, eventual collapse, indicating that the remaining effective dielectric charge is insufficient to sustain the induced junction. To maintain junction functionality after UV exposure, shallow As- and Sb-implanted junctions are employed, resulting in an initial reduction during 222 nm exposure followed by stabilization at around 80–85% of the initial value up to the highest tested dose of 200 J/cm^2^. Further improvement is achieved by stripping and regrowing the implanted screen oxide before SiN_x_ deposition, yielding nearly unchanged photocurrent after prolonged 222 nm exposure up to ca. 500 J/cm^2^. These results show that UV stability can be substantially improved by reducing device dependence on dielectric-induced inversion and by improving post-implantation interfacial oxide quality.

## 1. Introduction

Induced-junction photodiodes based on dielectric surface passivation have emerged as promising candidates for high-accuracy optical radiometry and predictable quantum-efficient detectors [1,2,3,4]. By exploiting fixed positive charge in dielectric stacks such as SiO_2_/SiN_x_ to create a near-surface inversion layer in p-type silicon, these devices enable efficient carrier collection without a conventional diffused junction [3,5,6]. This principle forms the basis of Predictable Quantum Efficient Detectors (PQEDs), which can achieve near-unity internal quantum efficiency (IQE) and extremely low internal quantum deficiency (IQD), making them attractive for primary optical power standards and for calibration or monitoring of optical and ultraviolet sources in applications requiring stable and traceable radiometric measurements [3,5].

We previously demonstrated SiO_2_/SiN_x_-passivated induced-junction photodiodes with record-high quantum efficiency in the 400 nm to 700 nm range, achieving IQD below 10^−5^ across the visible spectrum [3]. These results confirmed the suitability of dielectric-induced junctions for high-precision radiometry in the visible. However, extending operation into the ultraviolet (UV) region introduces additional challenges, as carrier generation shifts closer to the surface and device performance becomes increasingly sensitive to dielectric stability and interface quality.

Wide-bandgap semiconductors such as AlGaN/GaN and SiC are attractive alternatives for UV photodetection, particularly for solar-blind and high-temperature applications, and extensive research has been devoted to such devices [7,8]. However, their wide band gap limits operation in the visible range. Silicon photodiodes remain important in high-accuracy optical radiometry because of their mature fabrication technology, well-understood responsivity, and compatibility with predictable quantum-efficient detector concepts. Since fast temporal response is not a primary requirement for the intended radiometric application, material selection here is driven more by spectral coverage, responsivity, and stability than by high-speed detector operation.

UV irradiation is known to modify dielectric materials through mechanisms such as charge trapping, bond breaking, and defect generation [9,10,11,12]. In PECVD SiN_x_, UV exposure can alter fixed charge density and increase interface-state density, thereby degrading surface passivation and modifying the induced electric field [11,12,13]. We have previously shown that adding a thin interfacial thermal SiO_2_ layer significantly lowers IQD despite the lower positive fixed charge of SiO_2_ compared with SiN_x_ [3]. This improvement arises from the superior interface quality of thermal SiO_2_, which substantially reduces interface defect density [14,15,16,17]. The SiN_x_ layer remains desirable, not only because of its high positive fixed charge [18], but because it serves as a hydrogen reservoir that can passivate dangling bonds at the Si/SiO_2_ interface [3,15]. In addition, its refractive index makes it useful as an antireflective coating. At the same time, SiO_2_ itself can exhibit UV-induced charge modification, particularly at shorter wavelengths [9,10], and the combined SiO_2_/SiN_x_ stack has been shown to exhibit wavelength- and dose-dependent degradation under UV [12,13]. However, for creating induced junctions on p-type silicon, there are currently no suitable UV-transparent alternatives to thermal SiO_2_ or SiN_x_, making the development of UV-stable passivation schemes important for applications in which p-type silicon is desired.

For induced-junction photodiodes fabricated on p-type silicon, the functionality of the device critically depends on maintaining sufficient positive fixed charge in the dielectric to sustain inversion at the surface. We have previously observed a collapse of photocurrent during UV exposure below 300 nm [13], indicating that the dielectric charge state becomes a limiting factor in the deep UV.

In the present work, we investigate the evolution of dielectric charge and passivation quality under UV exposure to better understand the degradation of SiO_2_/SiN_x_-based induced-junction photodiodes. We further examine whether shallow external implantation can improve stability at 222 nm by reducing device dependence on dielectric-induced inversion. Finally, we investigate the effect of stripping and regrowing the screen oxide after implantation, rather than thinning the screen oxide to the desired thickness to assess the role of oxide quality in UV degradation.

The results provide insight into the physical mechanisms governing UV-induced modification of dielectric stacks and clarify the role of junction engineering and oxide processing in extending the operational wavelength range of high-accuracy silicon photodiodes toward the deep UV region.

## 2. Materials and Methods

### 2.1. Device Fabrication

Three different structure categories were investigated in this study: (i) symmetrically passivated implanted and unimplanted silicon wafers for lifetime measurements, (ii) MIS capacitors for dielectric characterization, and (iii) photodiodes with either induced or shallow implanted junctions.

#### 2.1.1. Dielectric Stack

Dielectric stacks were prepared using 500 µm float-zone P-type (boron) Si (100) wafers from Topsil GlobalWafers A/S, Frederikssund, Denmark with resistivity of 5–12 kΩ∙cm.

All wafers were subjected to a HF dip, SC1 (NH_4_OH/H_2_O_2_/H_2_O), and SC2 (HCl/H_2_O_2_/H_2_O) treatments followed by DI water rinsing and spin drying.

Preparation of the basic induced junction stack consisted of ca. 6.5 nm thermal SiO_2_ grown at 1000 °C in a horizontal tube furnace, followed by ca. 65 nm PECVD SiN_x_ using a SiH_4_:NH_3_ flow ratio of 1:3. The development of this stack has been described previously in [3].

For shallow junction formation, selected wafers were implanted with As or Sb at Ion Beam Services (France) and furnace-annealed prior to dielectric stack formation. Implant and annealing parameters were selected based on profile simulations. A 30 nm thermal screen oxide was grown on these wafers prior to implantation. After annealing, the screen oxide on the symmetrically implanted test wafers was trimmed down to ca. 4 nm in a SC1 bath. The target thickness was 6 nm, but the etch was difficult to control due to the altered etch behaviour of the implanted oxide and changes in bath activity over time. A total of 65 nm SiN_x_ was then deposited using the same PECVD process. For photodiode production the screen oxide was instead trimmed down to ca. 15 nm prior to deposition of 26 nm PECVD SiN_x_. A summary of implantation and annealing conditions is provided in Table S1.

#### 2.1.2. MIS Capacitors

Wafers used for impedance measurements were heavily implanted with boron on the backside to form a low-resistance ohmic contact and were passivated only on the front side. Aluminium (150 nm) was deposited on both sides by thermal evaporation. Circular front-side electrodes (diameter: 1, 2 and 3 mm) were defined by shadow mask.

#### 2.1.3. Photodiodes

**Figure 1:**
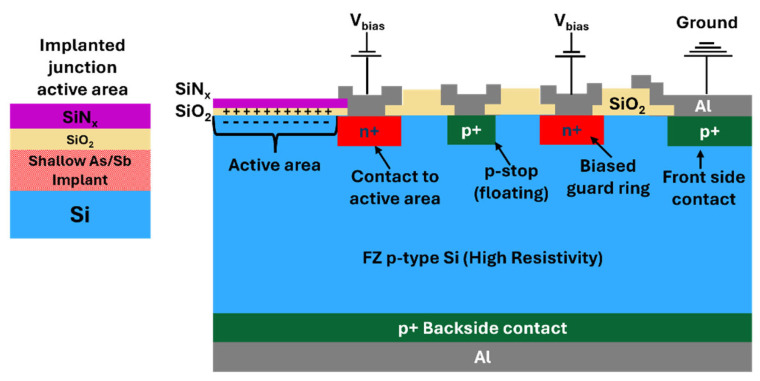
Cross-sectional schematic of the photodiode structure used in this work, showing the induced-junction (inversion-layer) active area and the biasing of the contact ring and guard ring. The n^+^ regions (phosphorus implant) provide electrical contact to the induced junction and guard ring, while p^+^ regions (boron implant) form the p-stop and substrate contacts. The active area is passivated by a thin interfacial SiO_2_/SiN_x_ stack, whereas the oxide separating contact regions is a thicker field oxide. The + and − symbols above the active area schematically indicate the dielectric charge and the corresponding induced inversion layer at the silicon surface. For implanted-junction variants, illustrated on the left, the active area received an additional shallow n-type (As or Sb) implantation before passivation. Reproduced based on the design reported in [3].

Photodiodes of active areas of 11 mm × 11 mm, and 11 mm × 22 mm were included in the wafer layout. A diagram of the layout is presented in Figure 1. The design included an n^+^ ring for contacting the active area, a p-stop ring for isolation, an n^+^ guard ring to suppress edge leakage, and a p^+^ ring at the periphery for top-side contact to the substrate.

For the photodiode fabrication, the same wafer types were included as was used for preparing the dielectric stacks. In addition, a few diodes were prepared on 500 µm FZ, P-type Si (111) wafers with >5 kΩ∙cm from Global Net Corp, Tokyo, Japan.

A thick thermal SiO_2_ layer was first grown to serve as field oxide during processing. The wafers were processed using six photomask layers that were used to define the areas of p^+^ implantation, n^+^ implantation, shallow As/Sb doping, active area dielectric passivation, contact holes through SiO_2_, and metallization. The p^+^ and n^+^ electrodes were made with boron and phosphorus implantation, respectively. Multiple implantation and dielectric stack variations were evaluated for the active area. A complete list of all tested variations is presented in Table S2. The wafers were metallized with 1.2 µm Al on both sides. 

### 2.2. Methodology

Oxide and nitride thicknesses were determined by spectroscopic ellipsometry in the 371 nm to 1000 nm range using standard optical models for thermal SiO_2_ and SiN_x_.

Capacitance–voltage (C–V) measurements were performed using a Signatone S-1060R manual probe station (Signatone, Gilroy, CA, USA) connected to a Keithley 4200 Semiconductor Characterization System (Keithley Instruments, Solon, OH, USA) in parallel conductance mode at 1 kHz with an AC amplitude of 100 mV, using bidirectional voltage sweeps. Samples were briefly illuminated prior to each sweep to remove trapped-charge effects, and all measurements were conducted in darkness. The presented C–V curves correspond to sweeps from inversion toward accumulation.

The flatband voltage was determined from the C–V curves, and the effective charge density, *Q*_eff_, was calculated from the flatband shift using the measured oxide capacitance. The bidirectional measurements indicated negligible contributions from mobile charge in the accumulation region. However, the extracted *Q*_eff_ should be interpreted as an effective net dielectric charge density inferred from the flatband shift under the present measurement conditions, rather than as a unique measure of one specific charge species. The metal–semiconductor work function difference was calculated individually for each sample based on the substrate doping concentration extracted from the depletion region (1/*C*^2^ − *V*) slope. Series resistance correction was evaluated and found to have negligible impact at 1 kHz; therefore, no correction was applied to the presented data.

The injection-dependent effective minority carrier lifetime was measured at 25 °C using Sinton WCT-120 systems (WCT-120 and WCT-120TS) (Boulder, CO, USA) operated in transient photoconductance decay (PCD) mode. All reported lifetime values correspond to an excess carrier concentration of Δ*n* = 5 × 10^14^ cm^−3^. Agreement between the two instruments was verified at this injection level. The surface saturation current density, *J*_0s_, was extracted using the Kane–Swanson slope method [19], using an intrinsic carrier concentration of 8.305 × 10^9^ cm^−3^ at 298.15 K.

Photoluminescence images were acquired using a BT Imaging LIS-R1 system (BT Imaging Pty Ltd., Waterloo, NSW, Australia) with 808 nm excitation. The PL intensity was calibrated to effective minority carrier lifetime using a reference measurement obtained in the central region of each sample. In the present work, the PL images are used for relative comparison of spatial lifetime variations before and after UV exposure. The absolute lifetime values derived from the PL images are therefore not used for quantitative analysis.

Implantation and annealing profile simulations were performed using Sentaurus Process from the Synopsys Advanced Technology Computer-Aided Design Suite (version W-2024.09-SP1) to guide the selection of screen oxide thickness, implant energies, doses, and annealing conditions for shallow junction formation. The simulated dopant distributions were subsequently compared with secondary ion mass spectrometry (SIMS) measurements performed by Eurofins EAG Laboratories on implanted and annealed wafers. Quantitative depth profiles of As and Sb were obtained, and depth calibration was performed by the service provider based on sputter crater measurements. The resulting profiles were used to determine dopant depth distribution and peak dopant concentration.

UV exposure was carried out using sources with nominal wavelengths of 300, 279, and 222 nm, with the corresponding normalized emission spectra presented in Figure S1. UV LEDs were used for irradiation at 300 and 279 nm, specifically theM300L4 from Thorlabs (Newton, NJ, USA) and the LEUVA66H70HF00 from LASER COMPONENTS Germany GmbH (Olching, Germany), respectively), while a UV222 excimer lamp from UVMedico A/S (Åbyhøj, Denmark) was used for the irradiation at 222 nm. Photodiode UV-exposure measurements were carried out at room temperature with an applied bias of 5 V. For wafer-level experiments, samples were positioned behind a 20 mm aperture, with a collimating tube placed between the UV source and the sample to define the illuminated area. For 222 nm exposure of the photodiodes, a mask with a circular aperture was attached directly to the device, resulting in an exposed area with a diameter of 8 mm. The irradiance at the photodiode surface was ca. 840 µW/cm^2^ and was recorded before and after each measurement with a calibrated reference detector. During the measurement campaign, the 222 nm lamp intensity increased by ca. 0.5% per day. UV stability was evaluated as a function of accumulated radiant exposure (hereafter referred to as dose). For dose calculations, the irradiance during each individual measurement was approximated by linear interpolation between the values recorded at the start and end of the run. The target dose was at least 70 J/cm^2^, however, some measurements were terminated early because of severe degradation, whereas others were extended because of promising UV stability. Photocurrent was recorded at 10 s intervals.

Spectral responsivity measurements were performed before and after UV exposure on quadrants of selected photodiodes with a sensitive area of 11 mm × 11 mm, using a spot diameter of 3.1 mm and with an applied reverse bias of 40 V. For each wavelength, the photocurrent of the sample was measured relative to that of a calibrated reference detector, and the responsivity change was evaluated from the ratio of the post-exposure and pre-exposure measurements, following established PTB procedures for spectral responsivity calibration of silicon photodiode-based detectors [20]. The reference-detector signal was corrected for temporal drift between the pre- and post-exposure measurement campaigns. One quadrant was left unexposed, while the remaining quadrants were exposed to different doses of 222 nm radiation. For wavelengths of 200–405 nm, the measurements were carried out using a xenon lamp and two 3-element reflection trap detectors with S5227-1010 and S1337-1010 photodiodes (Hamamatsu Photonics K.K., Hamamatsu City, Japan), while for wavelengths of 395–1000 nm, a tungsten-halogen lamp, one three-element reflection trap detector with an S6337 (Hamamatsu Photonics K.K., Hamamatsu City, Japan), and one six-element transmission trap detector with S1337 photodiodes were used. Trap detectors were used as high-accuracy transfer standards because their geometry strongly reduces reflectance losses compared with a single photodiode. A double-grating monochromator with order-sorting filters and a detector-positioning system was employed. The measurements were performed at 0° and 90° orientation to determine the spectral responsivity change for non-polarized radiation.

## 3. Results and Discussion

### 3.1. Preliminary Investigation of Induced-Junction Photodiodes

Induced-junction photodiodes previously fabricated using SiN_x_/SiO_2_ surface passivation were found to degrade under UV exposure at wavelengths of 279 nm and below [13]. To investigate the origin of this behaviour, MOS capacitors (MIS structures) were fabricated to quantify changes in effective dielectric charge as a function of UV wavelength and dose. In parallel, symmetrically passivated double-side polished wafers were prepared to evaluate the impact of UV exposure on effective minority carrier lifetime. For the unexposed wafers, the extracted surface saturation current density was 10 ± 4 fA/cm^2^.

**Figure 2:**
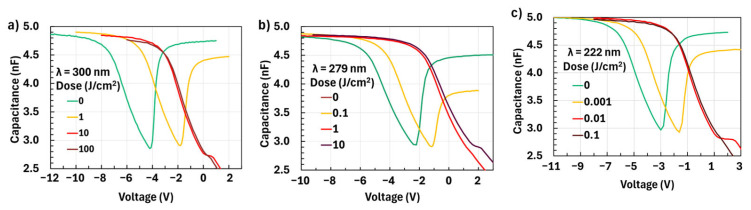
Capacitance–voltage characteristics at 1 kHz of MIS capacitors passivated with ca. 6 nm thermal SiO_2_ and ca. 65 nm PECVD SiN_x_ after UV exposure at the indicated doses for (**a**) 300 nm, (**b**) 279 nm, (**c**) 222 nm.

The C–V characteristics measured after exposure to different UV wavelengths and doses are shown in Figure 2. For all investigated wavelengths (300, 279, and 222 nm), a clear flatband voltage shift is observed with increasing UV dose. The extracted effective charge density, *Q*_eff_, decreases with increasing dose and approaches a saturation level beyond a characteristic exposure. The extracted effective charge and the corresponding lifetime on symmetrically passivated samples are summarized in Table 1.

Both the effective charge and the carrier lifetime decrease rapidly upon initial UV exposure, followed by saturation at higher doses. For 222 and 279 nm *Q*_eff_ stabilizes at approximately 3 × 10^11^ cm^−2^, whereas for 300 nm, saturation is reached at a higher level of ca. 7 × 10^11^ cm^−2^. In previous photodiode measurements [13], less than 0.4% photocurrent degradation was observed under 300 nm exposure. Taken together with the present MIS results, this suggests that an effective charge level on the order of 7 × 10^11^ cm^−2^ remains sufficient to sustain the induced inversion layer. In contrast, the reduction to ca. 3 × 10^11^ cm^−2^ at shorter wavelengths is consistent with the induced junction no longer being sufficiently sustained, resulting in loss of photocurrent.

The observed saturation behaviour suggests that UV exposure results in filling of a finite density of electrically active trap states in the dielectric stack, likely through electron trapping, thereby reducing the net positive dielectric charge, shifting the flatband voltage and weakening the surface field that supports the passivation. The accompanying degradation in passivation is reflected in an increase in *J*_0s_ (Figure S2), and a corresponding decrease in effective carrier lifetime. That the saturation level differs between 300 nm and the shorter wavelengths suggests that additional UV-activated charging pathways, or a larger fraction of electrically active states, become accessible at photon energies corresponding to 279 nm (4.4 eV). The similar saturation level observed at 279 nm and 222 nm further suggests that the dominant processes affecting the net charge evolution are already active at 279 nm under the present exposure conditions. The results are in agreement with previous studies reporting carrier injection from Si into the dielectric below certain wavelengths [12,21,22].

These results suggest that improved UV stability of induced-junction-based photodiodes may be achieved either by maintaining sufficient net positive dielectric charge after UV exposure or by reducing the density of UV-accessible electrically active states. Since implantation and subsequent oxide thinning can leave behind an implantation-affected interfacial oxide, replacing this oxide with newly grown thermal oxide is expected to improve the interfacial oxide quality and reduce the density of UV-accessible trap states. In the present study, these ideas are addressed through junction engineering and replacement of the interfacial oxide after implantation.

### 3.2. Development of Shallow As and Sb Doping Profiles

To reduce reliance on dielectric-induced inversion while maintaining efficient short-wavelength carrier collection, shallow n-type junctions were formed by ion implantation. Compared with phosphorus, heavier dopants such as As and Sb exhibit reduced projected range at a given implant energy because their larger mass leads to stronger nuclear stopping and greater energy loss during collisions with the silicon lattice. They also diffuse more slowly during annealing because of their lower mobility in silicon, making them attractive candidates for realizing shallow profiles [23].

A range of implant energies and doses for both As and Sb was first screened using implantation profile simulations, and the most promising conditions were then evaluated experimentally using furnace activation anneals (Table S1). Simulated profiles showed reasonable agreement with subsequent SIMS measurements (Figures S3 and S4). To maintain a shallow junction while limiting implantation-induced damage in silicon, the implant energies were chosen such that the concentration peak remained within the screen oxide and only the tail of the distribution extended into the silicon. The primary design target was to obtain peak dopant concentrations 10^19^–10^20^ cm^−3^ in silicon, while keeping the dopant distribution as close to the surface as possible and limiting concentrations to a range compatible with electrical activation [24].

**Figure 3:**
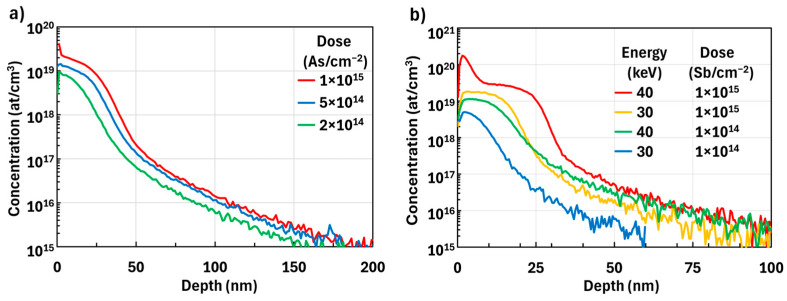
SIMS profiles of silicon wafers with 30 nm screening oxide and implanted following (**a**) As at 25 keV for the indicated doses and (**b**) Sb implantation at the indicated doses and energies. The wafers were annealed for 1 min at 900 °C.

Because spreading resistance profiling (SRP) proved insufficiently reproducible for these very shallow junctions, the implant development relied on a combination of SIMS depth profiling, sheet resistance measurements, and injection-dependent lifetime/*J*_0s_ extraction. Representative SIMS profiles for As implants (25 keV, varying dose) and Sb implants (varying energy and dose) after a 1 min, 900 °C activation anneal are shown in Figure 3. As expected, increasing implant dose primarily increased the near-surface concentration while maintaining a similar depth distribution, whereas Sb profiles showed a strong dependence on implant energy, with 40 keV producing a more pronounced near-surface peak compared with 30 keV at comparable dose.

**Figure 4:**
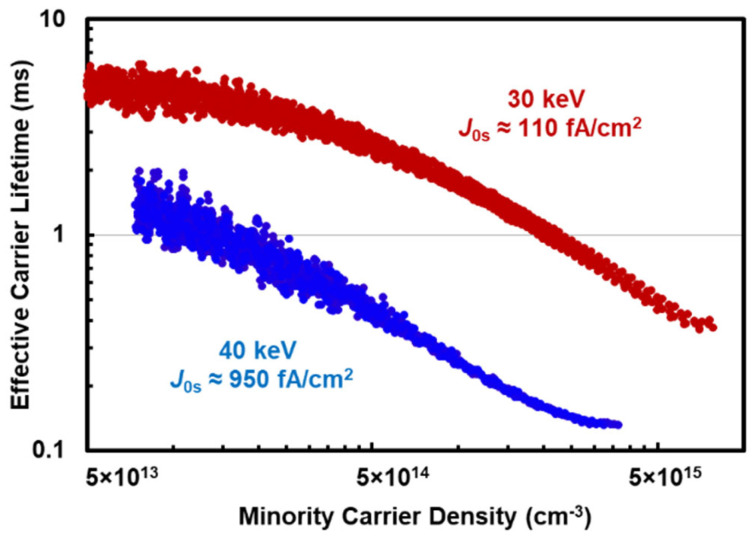
Photoconductance decay measurements on wafers implanted on each side with a dose of 1 × 10^15^ Sb/cm^−2^ at 30 and 40 keV and symmetrically passivated with ca. 4 nm of SiO_2_ and 65 nm SiN_x_ before UV exposure. Each wafer was measured at five different locations across the wafer.

Electrical passivation quality differed markedly between Sb implant energies. Wafers implanted with Sb at 40 keV exhibited significantly poorer surface passivation than those implanted at 30 keV, consistent with a higher near-surface defect density and/or incomplete electrical activation associated with the higher peak concentration. This is reflected in the injection-dependent lifetime behaviour and extracted surface saturation current density, where the 40 keV condition yielded substantially higher *J*_0s_ than the 30 keV condition (Figure 4). The extracted *J*_0s_ of ca. 110 fA/cm^2^ for the 30 keV wafer is also about an order of magnitude higher than that of the induced junction diodes. Assuming that the effective dielectric charge is of similar order to that in the induced-junction reference structures, the increased *J*_0s_ is consistent with increased interface-related recombination (e.g., higher *D*_it_ and/or implant-induced near-surface defects).

Based on these observations, selected Sb implant conditions (dose 1 × 10^15^ cm^−2^ at 30 and 40 keV) were carried forward for UV exposure studies on symmetrically passivated wafers. After double-sided implantation and activation (1 min at 900 °C), the screen oxide was thinned to approximately 4 nm and a ca. 65 nm PECVD SiN_x_ layer was deposited on both sides. The wafers were then diced into 4 × 4 cm^2^ samples for controlled UV exposure.

**Figure 5:**
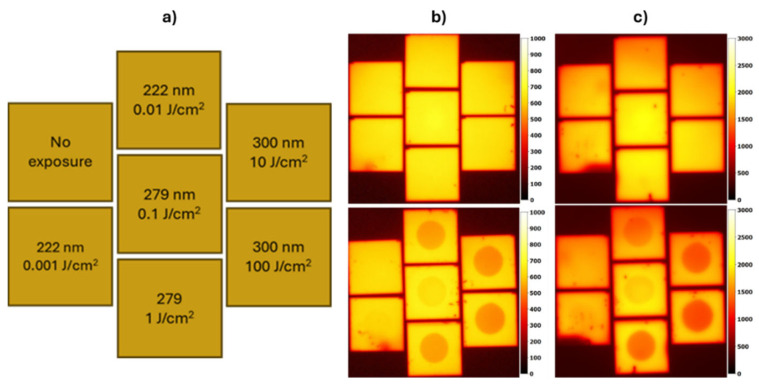
(**a**) Schematic overview of the individual sample pieces and their corresponding UV exposure conditions. (**b**,**c**) Photoluminescence images acquired before (top) and after (bottom) UV exposure for wafers implanted with Sb at a dose of 1 × 10^15^ Sb/cm^−2^ with implant energy of 40 keV (**b**) and 30 keV (**c**). The colour bar shows the effective minority carrier lifetime in µs.

To evaluate spatial non-uniformities and localized degradation, photoluminescence images were acquired before and after spot exposures under the conditions outlined in Figure 5a. Figure 5b,c show the photoluminescence images for the 40 keV and 30 keV Sb implants. A reduction in effective lifetime was observed in exposed regions for all tested implantation conditions, including at 300 nm. Compared with induced-junction samples exposed to the same doses, the implanted samples exhibited a larger relative lifetime reduction at 300 nm, suggesting that UV exposure produces a stronger increase in surface-related recombination in these structures. Possible contributors include a higher density of electrically active defects in the interfacial oxide after implantation and oxide thinning, changes in the surface potential that modify carrier recombination near the interface, and/or greater UV-induced defect formation at the Si/SiO_2_ interface. However, as lifetime reduction alone does not directly determine UV photodiode performance, photocurrent stability measurements were required to assess whether shallow implantation improves UV stability. The subsequent photodiode batch therefore included multiple junction and dielectric-stack variations, allowing the most promising shallow-implant concepts to be evaluated directly at the device level.

### 3.3. UV Stability of Fabricated Photodiodes with and Without External Doping

A batch of photodiodes was fabricated with multiple variations in dielectric stack and junction design (summarized in Table S2). The dielectric stack differed somewhat from the MIS and lifetime test structures discussed in Section 3.1; the latter were used primarily to identify general UV-induced trends in dielectric charge and passivation behaviour. Since these preliminary measurements showed that degradation was most severe at 222 nm, photodiode UV stability was evaluated at this wavelength as the most stringent test condition, by monitoring photocurrent during exposure and reporting the normalized photocurrent as a function of accumulated dose.

#### 3.3.1. Induced Junction

**Figure 6:**
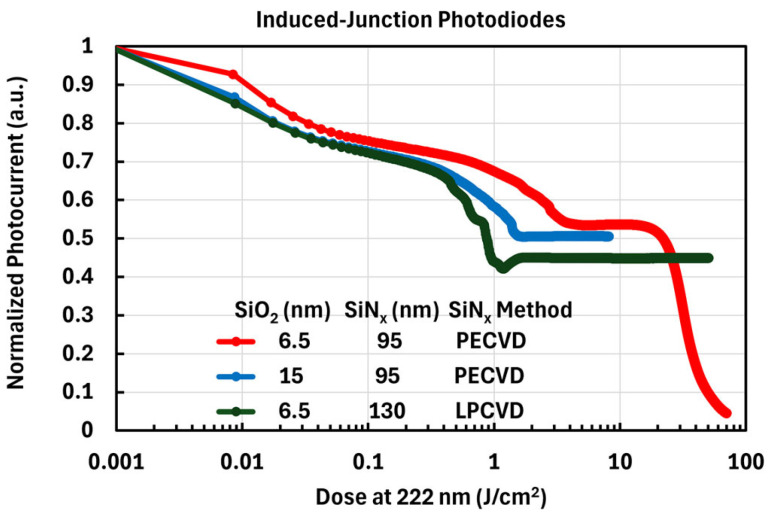
Normalized photocurrent vs. dose at 222 nm for induced-junction photodiodes with varying SiO_2_ thickness, SiN_x_ thickness, and SiN_x_ deposition method (note log scale).

A subset of the fabricated photodiodes was prepared as induced-junction devices, with only minor modifications relative to the previously reported SiO_2_/SiN_x_ structures [3]. Two variations were evaluated: (i) increasing the interfacial oxide thickness from 6.5 to 15 nm, and (ii) replacing PECVD SiN_x_ with a thicker LPCVD SiN_x_. The normalized photocurrent as a function of applied dose at 222 nm of the fabricated devices is presented in Figure 6. The two induced-junction devices with 100 nm PECVD SiN_x_ exhibited an initial photocurrent of approximately 30 µA under the applied measurement conditions, while it was approximately 23 µA for the one with 130 nm LPCVD SiN_x_, with the difference likely being due to differences in reflection and absorption. Upon 222 nm exposure, the photocurrent decreased rapidly by 25–30% within the first seconds of exposure for all samples, corresponding to a dose of 0.1 J/cm^2^, followed by a slower degradation toward a quasi-stable level of ca. 50% within approximately two hours.

Within the investigated range, increasing the interfacial oxide thickness to 15 nm did not produce a clear change in the 222 nm degradation behaviour.

For the devices employing a 6.5 nm interfacial SiO_2_ layer and a ca. 95 nm PECVD SiN_x_ cap, continued exposure led to a delayed collapse in photocurrent after ca. 10 h, consistent with earlier observations in similar structures [13]. The device with a thicker LPCVD SiN_x_ layer did not exhibit a comparable late-stage collapse within the measured dose range (up to 50 J/cm^2^), but more data would be required to determine whether this is representative behaviour.

The SiN_x_ deposition method can in principle influence both optical loss and interface quality, including through differences in film composition and intrinsic stress [25,26]. However, the three induced-junction photodiodes overall exhibited similar response to UV exposure, indicating that, within the limited set of variations investigated here, these changes did not substantially improve UV stability. This points to processes associated with the common Si/SiO_2_ interfacial region as a likely major contributor to the degradation. The observed behaviour is also qualitatively consistent with the results in Section 3.1, where the degradation was associated with a saturating reduction in effective net dielectric charge inferred from C–V measurements. UV-induced Si–H bond breaking in SiN_x_-based passivation has been reported previously [11,14], and may contribute here as well. However, the present results do not allow a unique separation of all microscopic degradation pathways. The charge-evolution picture nevertheless provides a simple and consistent framework for understanding the observed photocurrent loss.

Overall, these results show that the induced-junction variations investigated here did not substantially improve UV stability at 222 nm.

#### 3.3.2. Implanted Junction

**Figure 7:**
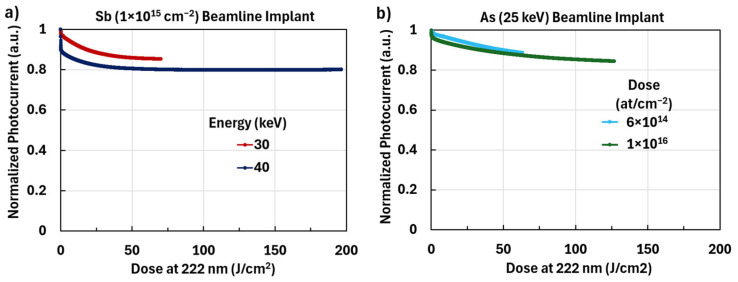
Normalized photocurrent vs. dose at 222 nm for photodiodes passivated with 15 nm SiO_2_ and 26 nm PECVD SiN_x_ and doped with (**a**) Sb, and (**b**) As.

Photodiodes incorporating shallow implanted junctions (Sb or As) were fabricated using a 15 nm SiO_2_/26 nm PECVD SiN_x_ stack. The normalized photocurrent as a function of applied dose at 222 nm of the fabricated devices is presented in Figure 7a (Sb) and Figure 7b (As). These devices exhibited a slightly lower initial photocurrent (ca. 25 µA) than the induced-junction photodiodes. Because the initial photocurrent was similar across all implanted variants, the difference is most plausibly explained by systematic optical effects (e.g., reflectance differences associated with dielectric thickness), rather than junction- or defect-related electrical losses. Similar to the induced-junction devices, and as expected based on the lifetime measurements, the implanted-junction photodiodes showed an immediate response during the first seconds of exposure, but the magnitude of the initial drop was <12%, as opposed to 25% to 30% for the induced junction devices.

In contrast to induced-junction photodiodes, both Sb- and As-implanted devices stabilized at significantly higher normalized photocurrent, approaching 80–85% of the initial value (corresponding to ca. 20 µA under the present measurement conditions). Sb-implanted photodiodes stabilized after receiving a dose of ca. 75 J/cm^2^, whereas As-implanted devices did not stabilize completely within the longest exposure time corresponding to a dose of 125 J/cm^2^. After exposure, all the externally implanted photodiodes delivered higher photocurrent than the induced-junction photodiodes under prolonged 222 nm exposure, despite their slightly lower initial photocurrent.

Overall, these results show that shallow implantation provides a clear improvement in UV stability relative to induced-junction devices. The improvement is consistent with reduced device dependence on dielectric-induced inversion, allowing carrier collection to be maintained more effectively as the dielectric charge state evolves during exposure.

### 3.4. Repairing Oxide After Implant

The preliminary results in Section 3.1 suggest that UV exposure reduces the effective positive charge in the dielectric stack through a saturating process, consistent with filling of a finite population of electrically active oxide/interface traps. For photodiodes incorporating shallow implants, additional implantation-related damage in the screen oxide and at the Si/SiO_2_ interface may further increase interface recombination and cause or accelerate UV-induced performance loss. These considerations motivate an approach in which the screen oxide is removed after activation and replaced by a regrown thermal oxide, thereby replacing the implantation-affected interfacial oxide with newly grown thermal oxide and potentially reducing the density of UV-accessible traps and/or interface states. The downside of this approach is that thermal regrowth of the oxide requires a high-temperature process that drives the dopants deeper into the silicon, causing the photogenerated carriers to require higher lifetime to reach the depletion region. A highly doped region near the surface is also often considered a “dead” layer with respect to charge collection. However, since UV photons are absorbed within the first few nanometres of silicon, it is not possible to make the doping layer so shallow that the UV photons do not interact with it. Consequently, it is more important to ensure that the carrier lifetime in the highly doped region remains sufficient for charge collection.

To evaluate the effect of regrowing the interfacial oxide, As-implanted photodiodes (25 keV, 1 × 10^16^ cm^−2^) were fabricated using two oxide process sequences following activation and prior to 26 nm PECVD SiN_x_ deposition: (i) retaining ca. 15 nm of the original screen oxide by thinning the oxide, and (ii) stripping the screen oxide and regrowing a ca. 23 nm thermal SiO_2_ layer. The regrown oxide thickness differed from the retained oxide due to the higher thermal oxidation rate of the highly doped surface making the final oxide thickness hard to predict. Since the As- and Sb-implanted photodiodes showed broadly similar UV-stability behaviour in the initial comparison (Figure 7), the study on the effect of oxide-regrowth was carried out only for the As-implanted process. As discussed in the previous section, variations in oxide thickness within this general range did not produce a clear trend in the induced-junction devices studied here, suggesting that the improved behaviour of the regrown-oxide device is unlikely to arise from oxide thickness alone.

**Figure 8:**
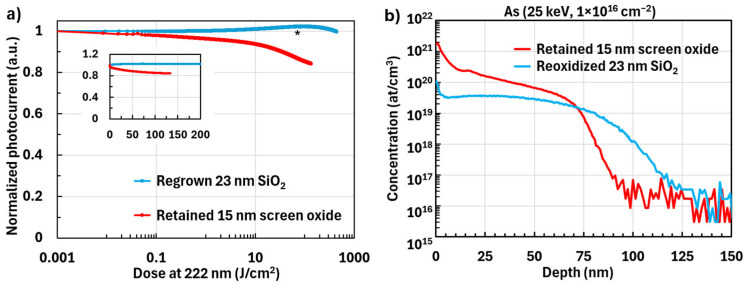
(**a**) Normalized photocurrent as a function of accumulated dose at 222 nm for As-implanted samples (25 keV, 1 × 10^16^ cm^−2^) fabricated with either retained 15 nm screen oxide (red) or stripped and regrown 23 nm thermal SiO_2_ prior to 26 nm SiN_x_ deposition (blue). The inset shows the initial 200 J/cm^2^ on a linear dose scale. * For the regrown-oxide sample, the data above approximately 72 J/cm^2^ were acquired in a separate follow-up measurement and aligned to the end of the initial run for plotting. (**b**) SIMS profiles of total As in Si for the corresponding devices after stripping the dielectric stack.

The normalized photocurrent during 222 nm exposure for the two oxide process sequences is shown in Figure 8a, while the corresponding SIMS profiles after removal of the dielectric stack are shown in Figure 8b. The device fabricated with regrown oxide exhibited a slightly lower initial photocurrent (ca. 22 µA) than the retained-oxide device (ca. 25 µA), which is consistent with increased optical losses expected from the thicker oxide and associated changes in reflectance. However, the regrown-oxide device showed substantially improved stability under prolonged 222 nm exposure. After approximately six days of exposure (corresponding to ca. 500 J/cm^2^), the photocurrent remained close to its initial level (ca. 22 µA) and exceeded the stabilized photocurrent of the retained-oxide device (ca. 20 µA) despite the lower starting value. Because of the strong stability observed in the regrown-oxide sample, the measurement was extended in a separate follow-up run to higher accumulated dose, as indicated in Figure 8a.

A minor initial increase in photocurrent (ca. 2%) was observed during the early stage of exposure before a gradual decrease for the photodiode with regrown oxide. The irradiance measured by the reference detector showed an approximately 0.5% increase after the initial dose of 72 J/cm^2^ and a 3% increase at the end of the six-day measurement, indicating that the initial rise in photocurrent might be partly associated with increasing irradiance over time, but cannot be explained by irradiance variation alone. This interpretation is also consistent with spectral responsivity measurements performed on different photodiodes from the same wafer before and after exposure to various doses at 222 nm (Figure S5), which showed a ca. 2% increase in responsivity at 222 nm for a dose of 100 J/cm^2^. The origin of this increase remains unclear, but a similar responsivity peak near 222 nm was also observed for the diode with retained screen oxide. Since these shortest wavelengths are most sensitive to the dielectric stack and the near-surface collection conditions, this common feature may reflect a UV-induced modification of the surface region, for example through changes in optical losses and/or carrier collection very close to the surface. The much larger and broader spectral change observed for the retained-oxide sample indicates that additional degradation mechanisms are present in that structure. Finally, it should be noted that the apparent late-stage decrease in the logarithmic-dose representation is partly influenced by axis compression; when plotted on a linear dose scale (inset in Figure 8a), the response is consistent with stabilization rather than accelerating degradation.

SIMS measurements (Figure 8b) confirmed that the strip-and-regrow process altered the near-surface dopant distribution relative to the retained-screen-oxide sample. The regrown-oxide sample exhibited a lower measured near-surface concentration and a broader profile extending deeper into the silicon, consistent with oxidation-driven consumption of the most heavily doped surface region and additional thermal redistribution of As during reoxidation. Despite the broader near-surface dopant distribution in the regrown-oxide sample, the devices showed markedly improved UV stability. This indicates that the improvement cannot be understood simply in terms of making the junction as shallow and sharply peaked as possible and instead points to an important role of the oxide/interface condition after implantation.

### 3.5. Summary

Previous work showed that SiO_2_/SiN_x_-passivated induced-junction photodiodes retain stable photocurrent under 300 nm irradiation, but degrade strongly at shorter UV wavelengths [13]. The present C–V and carrier-lifetime measurements clarify this wavelength dependence by showing that UV exposure reduces both the effective dielectric charge density and the effective carrier lifetime. At 300 nm, Q_eff_ and lifetime decrease initially and then saturate above approximately 10 J/cm^2^, with the effective charge density stabilizing at about 7 × 10^11^ cm^−2^. Since induced-junction photodiodes remain stable under 300 nm exposure, this indicates that this charge level is sufficient to maintain the induced junction. At 222 and 279 nm, however, Q_eff_ saturates at a substantially lower level of about 3 × 10^11^ cm^−2^, indicating that additional electrically active trap states become accessible at these wavelengths. The induced-junction photocurrent data show that this lower effective charge is insufficient to sustain the junction, leading to rapid loss of charge collection under 222 nm irradiation.

To maintain a junction during 222 nm exposure, suitable implantation and annealing conditions were first identified for shallow n-type As and Sb junctions. Photodiodes fabricated using these junction concepts still exhibited a rapid initial degradation, but then stabilized at approximately 80–85% of their initial photocurrent and remained stable at this level up to the highest tested dose of 200 J/cm^2^. The implant peak was intentionally placed in the screen oxide rather than in the silicon in order to keep the junction shallow. This likely increased the sensitivity of the Si/SiO_2_ interface to implantation-related damage and contributed to elevated surface recombination and reduced charge collection.

To recover the low interface state density associated with the thermal SiO_2_/Si interface, the screen oxide was stripped after implantation and activation and then regrown prior to SiN_x_ deposition. Despite the deeper profile due to the additional thermal treatment, this resulted in a clear improvement in UV stability, with the photocurrent remaining nearly unchanged after prolonged 222 nm exposure up to approximately 500 J/cm^2^. Although the resulting photodiodes are not completely immune to UV-induced changes, the results clearly show that shallow implanted junctions combined with regrown thermal oxide can be used to improve the UV stability of SiO_2_/SiN_x_-based photodiodes.

## 4. Conclusions

This work shows that UV degradation in SiO_2_/SiN_x_-based induced-junction photodiodes at 222 nm is closely associated with reduction of effective dielectric charge and the resulting weakening or loss of surface inversion. The results indicate that the charge density remaining after 300 nm exposure is sufficient to sustain the induced junction, whereas the substantially lower saturation level reached at 222 and 279 nm is not. Consistent with this, induced-junction photodiodes exposed to 222 nm exhibit an immediate photocurrent drop of 25–30%, followed by degradation toward approximately 50% of the initial value, and some collapse completely upon prolonged exposure. To maintain the junction and enable charge carrier collection after UV exposure, we show that shallow As or Sb implants can be employed. Implanted-junction devices exposed to 222 nm radiation stabilized at around 80–85% of their initial photocurrent up to the highest tested dose of 200 J/cm^2^. Further substantial improvement is achieved by stripping the implanted screen oxide and regrowing thermal oxide before SiN_x_ deposition. This resulted in nearly unchanged photocurrent during prolonged 222 nm exposure up to approximately 500 J/cm^2^, clearly outperforming devices fabricated with retained screen oxide. The study shows that shallow implanted junctions combined with regrown thermal oxide provide a promising route toward UV-stable silicon photodiodes for precision radiometric applications.

## Figures and Tables

**Table 1:** Evolution of effective charge density *Q*_eff_ and average effective carrier lifetime (at an excess carrier concentration of 5 × 10^14^ cm^−3^) following UV exposure with peak wavelengths of 222, 279, and 300 nm for the indicated doses. The unexposed sample is included as a reference. “NM” denotes not measured. Representative photoconductance decay and *J*_0s_ data for each wavelength and dose condition are shown in Figure S2.

Peak Wavelength (nm)	Dose (J/cm^2^)	*Q*_eff_ (cm^−2^)	Average Lifetime (ms) at 5 × 10^14^ cm^−3^
No exposure	0	(2.5 ± 0.3) × 10^12^	12.9 ± 3.3
222	0.001	1.6 × 10^12^	10.8
222	0.01	3.1 × 10^11^	4.7
222	0.1	2.9 × 10^11^	5.6
279	0.1	1.2 × 10^12^	8.5
279	1	2.8 × 10^11^	4.5
279	10	2.8 × 10^11^	5.1
300	0.01	NM	8.5
300	1	1.4 × 10^12^	NM
300	10	8.0 × 10^11^	7.8
300	100	7.0 × 10^11^	7.7

## Data Availability

The data supporting the findings of this study are openly available in Zenodo at https://doi.org/10.5281/zenodo.20426248.

## Supplementary Materials

The following supporting information can be downloaded at: https://www.mdpi.com/article/10.3390/s26133991/s1, Figure S1: Normalized emission spectra of the UV sources used for exposure experiments with peak wavelengths of 222, 279, and 301 nm. The source with a peak at 301 nm has a nominal wavelength of 300 nm and is referred to as having a peak wavelength of 300 nm in the main paper. Table S1: Implantation and annealing parameters evaluated for obtaining shallow n^+^ profiles using furnace activation. These data summarize implant and annealing conditions evaluated by 4-point probe and SIMS during junction optimization. Conditions for UV stability testing were selected based on evaluation of the resulting SIMS profiles and the measured sheet resistivity. Table S2: Overview of parameters used for the photodiode production. “NA” denotes “Not Applicable”. The listed SiN_x_ thicknesses correspond to the final thickness. The as-deposited layers were thicker, but their thickness was reduced during subsequent cleaning/process steps prior to completion of the devices. Figure S2: Representative injection-dependent effective carrier lifetime measured by photoconductance decay for symmetrically passivated wafers after exposure to the indicated doses at (a) 222 nm, (c) 279 nm, and (e) 300 nm. The corresponding Auger-corrected inverse lifetime plots are shown in (b), (d), and (f), respectively. For clarity, one representative dataset is shown for each condition. The surface saturation current density J_0s_ indicated in the plots was extracted from the slope of the linear fits by the Kane–Swanson slope method [19]. Figure S3: SIMS profiles for As-implanted wafers through a 30 nm screen oxide and 1 min furnace annealing. Dashed lines show the corresponding TCAD simulated profiles for total As. Figure S4: SIMS profiles for wafers implanted with 1 × 10^15^ Sb/cm^2^ through a 30 nm screen oxide and (a) with varying implant energy and 10 min furnace annealing, and (b) 30 keV implant energy and varying furnace annealing durations. Dashed lines show the corresponding TCAD simulated profiles for total Sb. Figure S5: Spectral responsivity ratio *S*_after_/*S*_before_, for photodiodes with retained screen oxide (a) and regrown oxide (b) after exposure to the indicated 222 nm doses. The pre- and post-exposure spectral responsivity measurements were separated by approximately four months. Measurements were performed on photodiodes from the same wafer as the devices shown in Figure 8 of the main text. Two light sources were used to cover complementary wavelength ranges: open circles denote data acquired using a tungsten-halogen lamp (395–1005 nm), while filled circles denote data acquired using a xenon arc lamp (198–405 nm).
