# Supplementary material for: Improving UV Stability of SiO2/SiNx-Passivated Silicon Photodiodes Through Shallow Junction Implantation and Oxide Regrowth
Source: Sensors (Basel). 2026 Jun 24;26(13):3991. doi: 10.3390/s26133991 (PMC13364108; doi:10.3390/s26133991)
Supplement: Supplementary file 1 [file sensors-26-03991-s001.zip › sensors-4372294-supplementary.pdf]

Supplementary Material

for "Improving UV Stability of SiO_2/SiN_x-Passivated Silicon Photodiodes Through Shallow Junction Implantation and Oxide Regrowth"

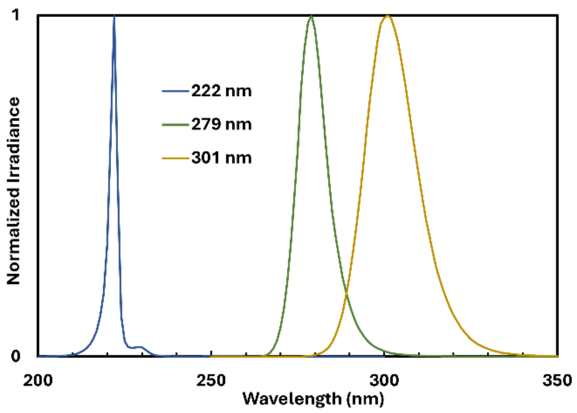

Figure S1: Normalized emission spectra of the UV sources used for exposure experiments with peak wavelengths of 222, 279, and 301 nm. The source with a peak at 301 nm has a nominal wavelength of 300 nm and is referred to as having a peak wavelength of 300 nm in the main paper.

Table S1: Implantation and annealing parameters evaluated for obtaining shallow n^+ profiles using furnace activation. These data summarize implant and annealing conditions evaluated by 4-point probe and SIMS during junction optimization. Conditions for UV stability testing were selected based on evaluation of the resulting SIMS profiles and the measured sheet resistivity.

Implant species	Energy (keV)	Dose (at/cm^2)	Annealing Temperature (°C)	Annealing Duration (min)	Sheet Resistance 4-point probe (Ω/sq)
As	25	2×10^{14}	900	1	3 692
As	25	5×10^{14}	900	1	2 260
As	25	1×10^{15}	900	1	1 544
As	25	1×10^{16}	900	1	222
As	35	1×10^{15}	900	1	556
As	35	1×10^{16}	900	1	182
Sb	30	1×10^{14}	900	1	11 650
Sb	30	1×10^{15}	900	1	2 630
Sb	40	1×10^{14}	900	1	3 635
Sb	40	1×10^{15}	900	1	867
Sb	55	2×10^{15}	900	1	451
Sb	55	1×10^{16}	900	1	708
Sb	30	1×10^{15}	900	10	2 799
Sb	30	1×10^{15}	900	60	3 040
Sb	40	1×10^{15}	900	10	951
Sb	40	1×10^{15}	900	60	1 022

Table S2: Overview of parameters used for the photodiode production. “NA” denotes “Not Applicable”. The listed SiN_x thicknesses correspond to the final thickness. The as-deposited layers were thicker, but their thickness was reduced during subsequent cleaning/process steps prior to completion of the devices.

Implantation	Implant Species	Screen Oxide thickness (nm)	Energy (keV)	Dose (at/cm^2)	Passivation Process
Beamline	Sb	30	30	1×10^{15}	Anneal 900°C, 10 min, Leave ca. 15 nm screen oxide, 26 nm PECVD SiN_x
Beamline	Sb	30	40	1×10^{15}	Anneal 900°C, 10 min, Leave ca. 15 nm screen oxide, 26 nm PECVD SiN_x
Beamline	As	30	25	6×10^{14}	Anneal 900°C, 10 min, Leave ca. 15 nm screen oxide, 26 nm PECVD SiN_x
Beamline	As	30	25	1×10^{16}	Anneal 900°C, 10 min, Leave ca. 15 nm screen oxide, 26 nm PECVD SiN_x
Beamline	As	30	25	1×10^{16}	Strip and regrow 23 nm thermal SiO_2, 26 nm PECVD SiN_x
None	NA	NA	NA	NA	15 nm thermal SiO_2, 95 nm PECVD SiN_x
None	NA	NA	NA	NA	6.5 nm thermal SiO_2, 95 nm PECVD SiN_x
None	NA	NA	NA	NA	6.5 nm thermal SiO_2, 130 nm LPCVD Si_3N_4

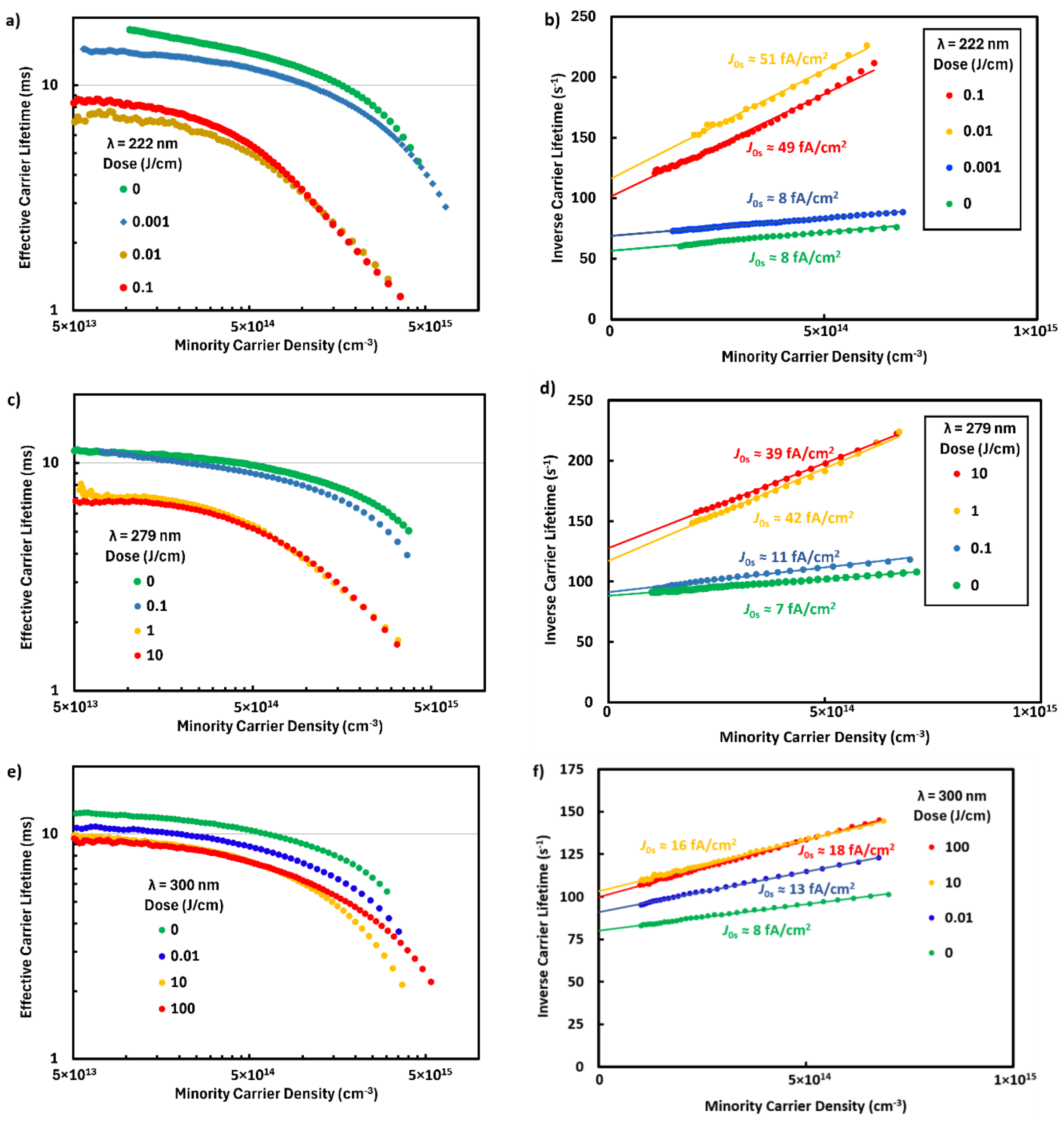

Figure S2: Representative injection-dependent effective carrier lifetime measured by photoconductance decay for symmetrically passivated wafers after exposure to the indicated doses at (a) 222 nm, (c) 279 nm, and (e) 300 nm. The corresponding Auger-corrected inverse lifetime plots are shown in (b), (d), and (f), respectively. For clarity, one representative dataset is shown for each condition. The surface saturation current density J_{0s} indicated in the plots was extracted from the slope of the linear fits by the Kane–Swanson slope method [1].

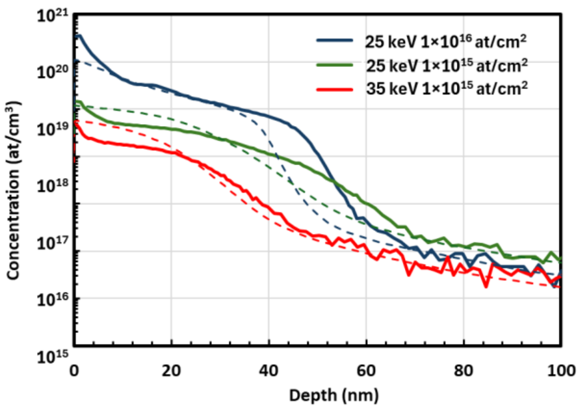

Figure S3: SIMS profiles for As-implanted wafers through a 30 nm screen oxide and 1 min furnace annealing. Dashed lines show the corresponding TCAD simulated profiles for total As.

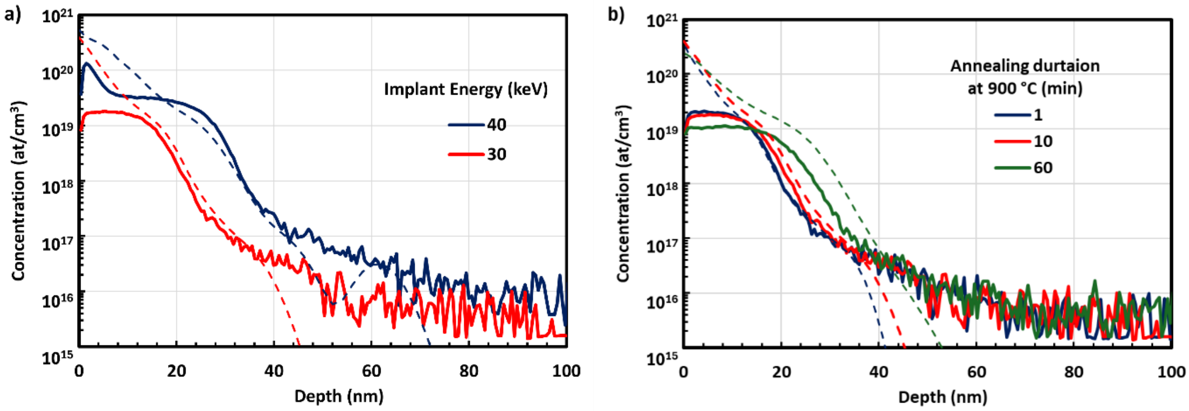

Figure S4: SIMS profiles for wafers implanted with 1×10^{15} Sb/cm^2 through a 30 nm screen oxide and (a) with varying implant energy and 10 min furnace annealing, and (b) 30 keV implant energy and varying furnace annealing durations. Dashed lines show the corresponding TCAD simulated profiles for total Sb.

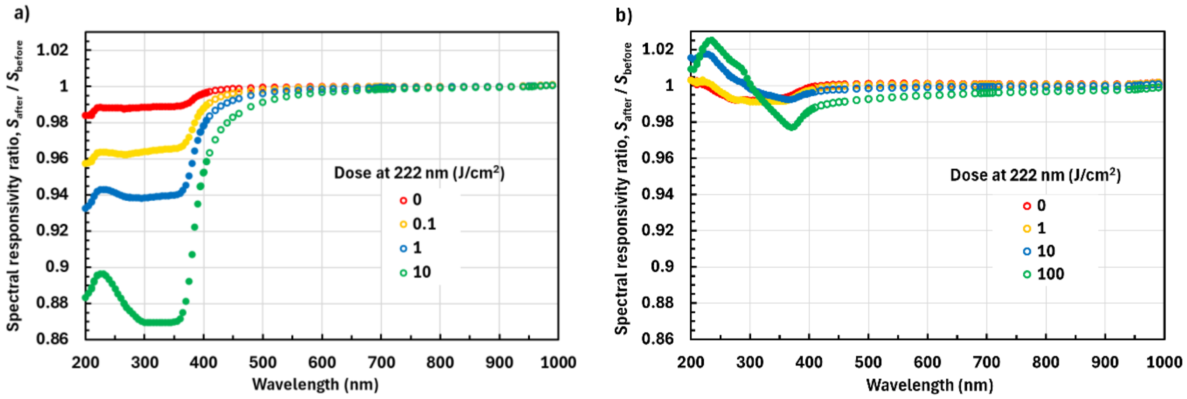

Figure S5: Spectral responsivity ratio S_{after}/S_{before}, for photodiodes with retained screen oxide (a) and regrown oxide (b) after exposure to the indicated 222 nm doses. The pre- and post-exposure spectral responsivity measurements were separated by approximately four months. Measurements were performed on photodiodes from the same wafer as the devices shown in Figure 8 of the main text. Two light sources were used to cover complementary wavelength ranges: open circles denote data acquired using a tungsten-halogen lamp (395-1005 nm), while filled circles denote data acquired using a xenon arc lamp (198-405 nm).

[1] D. E. Kane and R. M. Swanson, ‘Measurement of the emitter saturation current by a contactless photoconductivity decay method’, in *18th IEEE photovoltaic specialists conference, Las Vegas*, IEEE, New York, 1985, pp. 578–583.
